# Discrimination of Apple Liqueurs (*Nalewka*) Using a Voltammetric Electronic Tongue, UV-Vis and Raman Spectroscopy

**DOI:** 10.3390/s16101654

**Published:** 2016-10-09

**Authors:** Magdalena Śliwińska, Celia Garcia-Hernandez, Mikołaj Kościński, Tomasz Dymerski, Waldemar Wardencki, Jacek Namieśnik, Małgorzata Śliwińska-Bartkowiak, Stefan Jurga, Cristina Garcia-Cabezon, Maria Luz Rodriguez-Mendez

**Affiliations:** 1 Department of Analytical Chemistry, Faculty of Chemistry, Gdansk University of Technology, Narutowicza St. 11/12, 80-233 Gdańsk, Poland; m.e.sliwinska@gmail.com (M.Ś.); tomasz.dymerski@gmail.com (T.D.); walwarde@pg.gda.pl (W.W.); jacek.namiesnik@pg.gda.pl (J.N.); 2 Department of Inorganic Chemistry, Engineers School, Universidad de Valladolid, 47011 Valladolid, Spain; celiagarciahernandez@gmail.com; 3 The NanoBioMedical Centre, Adam Mickiewicz University, Umultowska 85, 61-614 Poznań, Poland; mikolaj.koscinski@amu.edu.pl (M.K.); msb@amu.edu.pl (M.Ś.-B.); stjurga@amu.edu.pl (S.J.); 4 Faculty of Physics, Adam Mickiewicz University, Umultowska 85, 61-614 Poznań, Poland; 5 Department of Materials Science, Engineers School, University of Valladolid, Valladolid 47011, Spain; crigar@eii.uva.es

**Keywords:** electronic tongue, voltammetric sensor, UV-Vis, Raman spectroscopy, apple liqueurs, *nalewka*

## Abstract

The capability of a phthalocyanine-based voltammetric electronic tongue to analyze strong alcoholic beverages has been evaluated and compared with the performance of spectroscopic techniques coupled to chemometrics. *Nalewka* Polish liqueurs prepared from five apple varieties have been used as a model of strong liqueurs. Principal Component Analysis has demonstrated that the best discrimination between liqueurs prepared from different apple varieties is achieved using the e-tongue and UV-Vis spectroscopy. Raman spectra coupled to chemometrics have not been efficient in discriminating liqueurs. The calculated Euclidean distances and the k-Nearest Neighbors algorithm (kNN) confirmed these results. The main advantage of the e-tongue is that, using PLS-1, good correlations have been found simultaneously with the phenolic content measured by the Folin–Ciocalteu method (R^2^ of 0.97 in calibration and R^2^ of 0.93 in validation) and also with the density, a marker of the alcoholic content method (R^2^ of 0.93 in calibration and R^2^ of 0.88 in validation). UV-Vis coupled with chemometrics has shown good correlations only with the phenolic content (R^2^ of 0.99 in calibration and R^2^ of 0.99 in validation) but correlations with the alcoholic content were low. Raman coupled with chemometrics has shown good correlations only with density (R^2^ of 0.96 in calibration and R^2^ of 0.85 in validation). In summary, from the three holistic methods evaluated to analyze strong alcoholic liqueurs, the voltammetric electronic tongue using phthalocyanines as sensing elements is superior to Raman or UV-Vis techniques because it shows an excellent discrimination capability and remarkable correlations with both antioxidant capacity and alcoholic content—the most important parameters to be measured in this type of liqueurs.

## 1. Introduction

Strong alcoholic beverages are complex mixtures of hundreds of components with different characteristics. The most common techniques used to analyze spirits and liqueurs made from apples are gas and liquid chromatography and spectroscopy. The analysis of spirit beverages made from apples has been recently reviewed [1,2]. Some of the most important parameters used for the characterization and differentiation of fruit liqueurs are the alcoholic content and the polyphenolic content [3].

Due to the complexity of the mixtures, a variety of multivariate data analysis techniques can be applied to data treatment in order to discriminate (for instance Principal Component Analysis or Cluster analysis), to classify the samples (using, among others, K-nearest neighbors) or to establish correlations with chemical parameters measured using classical techniques (using for instance Partial Least Square analysis) [4]. Spectroscopic sensors (UV-Vis, near infrared spectroscopy (NIR), Fourier transform infrared spectroscopy (FTIR), Raman or fluorescence) combined with multivariate data analysis are frequently used for beverage analysis [5]. While UV-Vis and fluorescence are limited to a small number of molecules, vibrational spectroscopy techniques are probing molecular vibrations present in many chemical species [6].

Analyzing strong alcoholic beverages with electronic tongues (e-tongue—also called taste sensors) can offer an alternative to spectroscopic sensors. An e-tongue is an instrument formed by a sensor array combined with mathematical procedures for signal processing based on multivariate data analysis [7,8,9,10]. E-tongues based on electrochemical sensors are advantageous because electrochemical sensors are cheap, portable and can be tailored to obtain complementary information that cannot be obtained by means of spectroscopy [5,11].

Potentiometric and voltammetric electronic tongues have been used to analyze a variety of foods and beverages [9,10,12,13], including drinks with low alcoholic content such as wines [14,15,16,17] or beers [18]. Regarding beverages prepared from apples, recently an e-tongue based on glassy carbon electrodes has been successfully used to compare taste profiles of ciders produced with different yeasts or to discriminate Polish commercial ciders [19]. Only few works have reported their use in the analysis of spirits with high alcoholic content such as vodka or whisky [20,21]. In those works, a potentiometric electronic tongue was used to evaluate the alcoholic content in strong alcoholic beverages. Since these early works, the voltammetric e-tongue technology has progressed, and new strategies to develop voltammetric sensors can be used to efficiently detect the alcohol content of spirits and liqueurs while keeping the sensitivity towards other compounds of interest [10,11].

The aim of this work was to evaluate the capability of a purposely designed voltammetric electronic tongue based on metallo-phthalocyanines (MPc) to analyze strong alcoholic beverages. The performance of the e-tongue was tested in *nalewka* liqueurs (a traditional Polish beverage) prepared from different varieties of apples. The discrimination capability and correlation with the alcoholic degree and the phenolic content (two of the main markers of the *nalewkas*’ quality) were also evaluated using UV-Vis and Raman spectra combined with chemometrics. The curves obtained from the three methods were preprocessed using variable reduction methods and the data obtained were used as the input variable for Principal Component Analysis (PCA). In addition, outputs of PCA were used to calculate Euclidean distances (E. distance) and coefficients of variation (CV). Application of the k-Nearest Neighbors algorithm (kNN) was used to confirm the discrimination capability results obtained from PCA and to classify the samples. Using Partial Least Squares (PLS-1), correlations were established with the alcoholic content measured by means of density and the polyphenolic content obtained using the Folin–Ciocalteu method, and the performance of the three methods has been compared.

## 2. Materials and Methods

### 2.1. Reagents and Solutions

All chemicals and solvents were of reagent grade and used without further purification. The solutions were obtained by dissolving substances in deionized water (resistivity of 18.2 MΩ/cm) obtained from a Milli-Q system (Millipore, Billerica, MA, USA).

Gallic acid (97.5%–102.0%), sodium carbonate (anhydrous, powder, 99.99%), Folin–Ciocalteu reagent, and ethanol (absolute, ≥99.8%, GC) were purchased from Sigma-Aldrich. Cobalt(II), iron(II) and zinc(II) phthalocyanines (CoPc, FePc, ZnPc) were also purchased from Sigma-Aldrich. Graphite ultra “F” purity was purchased from MERSEN USA.

### 2.2. Apple Nalewka Samples

*Nalewkas* are homemade liqueurs made almost exclusively from three ingredients: fruits or herbs, grain alcohol and sugar. The alcoholic strength of the final product is of ca. 35%–60% v/v [22]. Apple *nalewkas* used in this study were produced in homemade conditions using only apples, sugar, spirit 95% v/v and deionized water.

Five apple varieties were used to prepare *nalewkas*: Ligol, Kosztela, Grey Reinette, Rubin and Cox Orange. All varieties of apples were harvested in October 2014 in Pomeranian voivodship in Poland. Ligol and Kosztela varieties of apples originate from Poland, being the Kosztela, the oldest variety cultivated in Poland since XVIIth century. Ligol is a new variety developed at the Research Institute of Horticulture in Skierniewice in Poland [23]. The other three varieties originated from other European countries (Grey Reinette from France, Rubin from Czech Republic and Cox Orange from UK) but were widely cultivated in Poland [24].

After harvest, apples were thoroughly washed and chopped with the skin left on. A total of 225 g of each variety was placed in the corresponding jar and covered with sugar (75 g). Then, jars were sealed tightly and kept closed at an ambient temperature for 3 days. The next step was the addition of spirit (80 mL) and deionized water (50 mL). Jars were closed again with a screw cap and left in a dark and warm place (25 °C) for eight weeks. Three replicas per sample were prepared and analyzed.

### 2.3. Electronic Tongue, Raman Spectroscopy and UV-Vis Analysis

The electronic tongue consisted of an array of four carbon paste electrodes (CPEs) prepared using a previously published method [25]. The array included an unmodified carbon paste electrode (C-CPE) and three electrodes chemically modified with cobalt, iron and zinc phthalocyanines (denoted as CoPc–CPE, FePc–CPE and ZnPc–CPE, respectively). Voltammograms were registered at room temperature in a potentiostat/galvanostat PGSTAT128 (Autolab Metrohm, Utrecht, the Netherlands) using a three-electrode configuration cell. The CPEs were used as working electrodes, the reference electrode was Ag|AgCl/KCl 3 mol/L and the counter electrode was a platinum wire. Cyclic voltammetry was carried out with a scan rate of 0.1 V/s in the potential range from −1.0 V to +1.0 V (vs. Ag|AgCl). Three repetitions per sample were measured.

Raman spectra were obtained using an in Via Ranishaw Raman Microscopy system (Ranishaw, Old Town, Wotton-under-Edge, UK) with a 633 nm He/Ne laser (0.75 mW laser power, Stage I) and 1800 g/mm grating. The laser light was focused on the sample with a 50×/0.75 microscope objective (LEICA). All Raman spectra were obtained from 450 to 4000 cm^–1^ using 20 s acquisition time. All spectra were corrected by using the WiRETM 3.3 software attached to the instrument. Measurement of peak positions was performed by using Lorentz profile at OriginPro 8.3 software (Northampton, MA, USA).

UV-Vis spectra were registered from 380 to 780 nm in a Shimadzu UV-1603 (Kyoto, Japan) spectrophotometer using 1 cm path length quartz cuvettes.

### 2.4. Data Preprocessing and Statistical Analysis

Voltammograms and Raman spectra were normalized and pre-processed by means of an adaptation of a data reduction technique based on ‘kernels’ using Matlab v2014b (The Mathworks Inc., Natick, MA, USA) [26]. Using this variable reduction method, 10 variables were obtained from each voltammogram or Raman spectrum. These data were used as input variables in statistical analysis.

Variable reduction of UV-Vis spectra was carried out using the CIELab coordinates (L*, a*, b*, X, Y, Z) calculated following the recommendations of the Commission Internationale of L’Eclairage for the CIE illuminant D65 and 10° standard observer conditions using a Shimadzu Color Analysis Software (Kyoto, Japan) [27]. These coordinates were used to calculate other color parameters: C*, h*, BI.

The statistical analysis was performed by using The Unscrambler v9.7 (CAMO Software AS, Oslo, Norway). A non-supervised multivariate method, the Principal Component Analysis (PCA) was used to evaluate the discrimination capability of each method. Euclidean distances (E. distance) and coefficients of variation (CV) between groups of samples were calculated using Microsoft Excel 2007. Partial Least Squares (PLS-1) was used to establish correlations between the results obtained from the holistic methods (e-tongue, Raman or UV-Vis spectra) and the chemical parameters obtained using classical chemical methods. The kNN results were obtained by a program written in Python 3.5 programming language (Python Software Foundation, Wilmington, DE, USA).

## 3. Results and Discussion

### 3.1. Analysis of Density and Phenolic Content

After having the samples in a dark and warm place (25 °C) for eight weeks, the phenolic content was estimated using the Folin–Ciocalteu procedure [28]. The density was measured by a Density Meter (DMA 38, Anton Paar) at 20 °C. Results obtained are collected in Table 1.

### 3.2. Analysis of Liqueurs Using an E-Tongue

**Figure 1:**
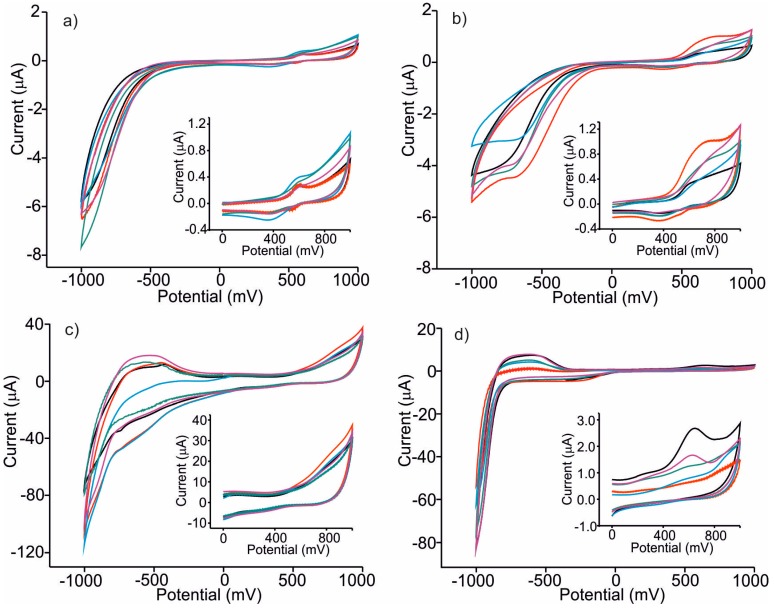
Cyclic voltammograms registered using CPEs immersed in *nalewkas* made from different varieties of apples. (**a**) Unmodified CPE; (**b**) ZnPc–CPE; (**c**) FePc–CPE; (**d**) CoPc–CPE Samples: Ligol (black), Kosztela (red), Grey Reinette (blue), Rubin (green), Cox Orange (purple).

Once prepared, the sensors were conditioned by performing repeatedly a cyclic voltammetry (five cycles) in a 0.1 mol/L KCl solution. This process allowed the obtainment of a stable voltammetric response before each measurement. After the conditioning step, the array of sensors was immersed in the *nalewka* samples diluted 1:10 in 0.1 M KCl and cyclic voltammograms were recorded. Figure 1 illustrates the response of each sensor forming the array towards *nalewka* samples prepared from different apple varieties. As observed in the figure, each electrode showed a particular response when immersed in apple *nalewka* liqueurs.

The electrochemical response obtained with C-CPE (Figure 1a) showed a redox process at positive potentials with the anodic peak at ca. 580 mV and the cathodic wave at 380 mV. Taking into account previous studies in alcoholic beverages [17,29], these peaks could be associated to the presence of polyphenols. Voltammograms also showed an intense cathodic peak at negative potentials that can be associated to the decomposition of the water/alcohol mixture. Voltammograms registered for the five *nalewkas* types showed similar habits, but differences in intensities could be observed from one type of sample to another due to their diverse composition.

Phthalocyanine-modified CPEs showed a variety of responses that arose from the dissimilar electrocatalytic effect of the three phthalocyanines used as modifiers and from the variety of compositions of *nalewka* liqueurs. It is interesting to highlight the strong electrocatalytic effect observed in CoPc–CPE and FePc–CPE electrodes that produced a clear increase in the intensity of the responses towards *nalewkas*. In particular, a broad anodic peak at ca. −800 mV could be observed in voltammograms registered using CoPc–CPE and FePc–CPE electrodes that, according to the literature, can be associated to the presence of organic acids (i.e., tartaric or malic) [30]. Moreover, the electrocatalytic effect towards the decomposition of the water/ethanol mixture produced a drastic increase in the intensity of the peaks at −1000 mV.

In summary, each sensor provides a unique response when immersed and cycled in an apple *nalewka*. Due to the interactions occurring between the electrode and the sample, the responses of a particular sensor differ from one apple, *nalewka,* to another (particularly in peaks related to phenols, organic acids and decomposition of water/ethanol). For these reasons, these electrodes, selected for this application, can be used to discriminate the liqueurs.

### 3.3. Analysis of Liqueurs Using UV-Vis and Color Analysis

**Figure 2:**
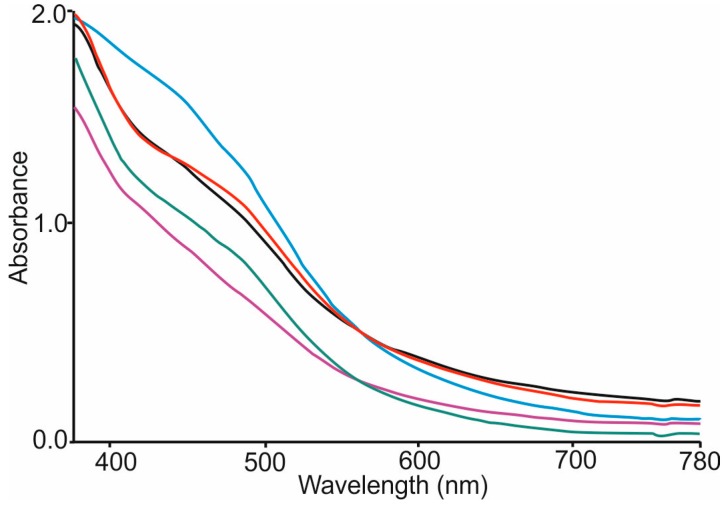
UV-vis spectrum of liqueurs made with different varieties of apple. Samples: Ligol (black), Kosztela (red), Grey Reinette (blue), Rubin (green), Cox Orange (purple).

UV-Vis spectra of the *nalewka* samples were registered from 380 to 780 nm. They were characterized by a broad band with the onset at 650 nm and a shoulder at ca. 500 nm (Figure 2). As the color of the liqueurs are related to their composition and in particular to the phenolic content, CIELab coordinates, were calculated from the UV-Vis spectra. Results are collected in Table 2.

Lightness (L*) values were similar in all *nalewka* samples, showing mean values of 87.05–92.91. The highest values of a* parameters were observed in *nalewkas* made from Kosztela and the lowest to Cox Orange apples (the negative value being related to green color and positive values with reddish tones). A sample made from Grey Reinette showed the highest b* positive values which respond to yellow tone. *Nalewkas* prepared from the Cox Orange variety had the smallest b* value.

The results of CIELab parameters were also used to calculate chroma C* (which reflects to color saturation), h* (Color hue angle) and browning index (BI). This parameter is usually used as an indicator of the browning extent in food products containing sugar. C*, h* and BI values showed by the beverage made from Cox Orange were clearly different from the rest and indicated that this *nalewka* did not have an intensive color. *Nalewka*s made from Grey Reinette appeared as the darkest and most intensive colored sample.

### 3.4. Analysis of Liqueurs Using Raman Spectroscopy

Vibrational spectroscopic techniques such as FTIR and Raman can be used to analyze food products [31,32]. Raman spectroscopy is more suitable for the analysis of aqueous systems because water has none or minimal interference with Raman scattering [33]. In addition, Raman spectra have been used to evaluate the alcoholic degree by establishing correlations between peak heights of ethanol and the alcoholic content for several alcoholic beverages [6,34,35].

**Figure 3:**
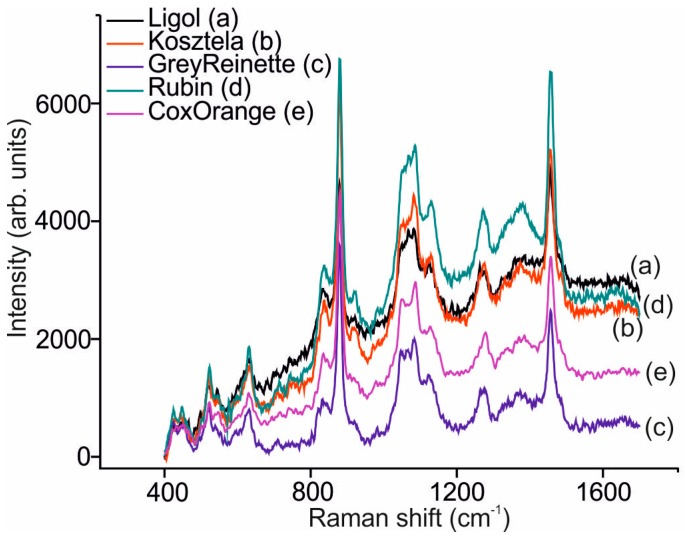
Raman spectrum of liqueurs made with different varieties of apple.

Figure 3 shows the Raman spectra of the apple, *nalewkas,* registered in the range of 400–1700 cm^−1^. As expected, in all *nalewka* samples, the dominating peaks were associated to the ethanol which is the majoritarian component of the sample. They included an intense peak at 879 cm^−1^ which is assigned to the C–C–O stretching vibration, and that is traditionally used to evaluate the alcoholic content of spirits; two small bands at 1045 cm^−1^ and 1085 cm^−1^ which are related to C–O stretching; and a peak at 1454 cm^−1^ assigned to the CH bending. The CH_2_ and CH_3_ stretching vibrations appear at 2877 cm^−1^, 2928 cm^−1^ and 2974 cm^−1^. Moreover, the broad band assigned to the O–H stretching (hydrogen bonds) appeared at 3252 and 3397 cm^−1^. These bands associated to ethanol were accompanied by other bands that were different in position and intensity from one *nalewka* to another. Moreover, the fluorescence background was also dependent on the apple variety used to prepare *nalewkas* and is also a feature that could help to discriminate the analyzed samples using chemometrics.

### 3.5. Statistical Analysis

The three sensing systems used in this work provided curves (voltammograms or spectra) showing a variety of peaks in different positions and with different intensities that can be used to discriminate the samples using chemometrics.

Usually, the initial step is to decrease the complexity of the system by reducing the number of variables (without loss of information). This new set of variables is then used as the input for a Principal Component Analysis (PCA), Euclidean distances (E.distance), the k-Nearest Neighbors algorithm (kNN) or Partial Least Squares 1 (PLS-1).

There are many solutions to simplify the high dimensionality of the curves, but it is important to use the most adequate for each situation. In the case of voltammetric electronic tongues, one solution is to use a feature extraction using the wavelet transformation [36], genetic algorithms [37] or using “kernels” [26,38]. In this paper, the Kernel method was used with the aim to obtain 10 variables from each voltammogram. In the case of UV-Vis spectra, a good approach is to reduce the number of variables by calculating the CIELab coordinates. In Raman or FTIR spectra, a common strategy for variable reduction is to use the first or second derivative of the curve. Using this method, the number of variables is still high (typically over 100 variables) [39]. In this work, the variable reduction of Raman spectra has been carried out using the Kernel method, in order to obtain a number of variables similar to that obtained using the e-tongue or the UV-Vis spectra in order to facilitate the comparison between different techniques.

**Figure 4:**
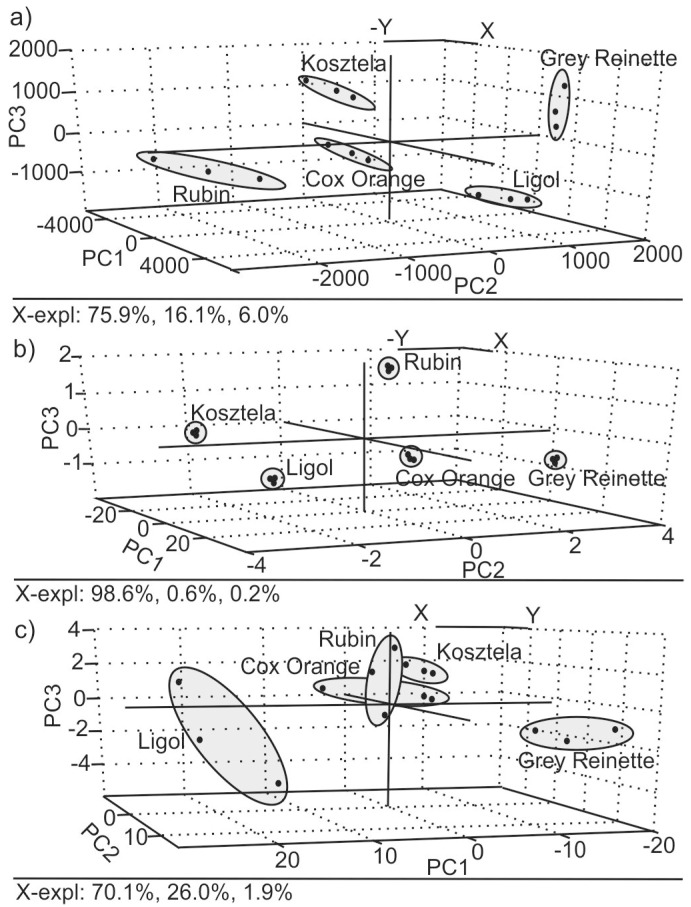
Principal component analysis scores plot corresponding to the classification of the five *nalewka* liqueurs using (**a**) e-tongue composed by an array of carbon paste electrodes modified with metal phthalocyanines; (**b**) using color parameters: L, a*, b*, C*, h* and BI; and (**c**) Raman spectra.

Figure 4a represents PCA score plots for the e-tongue using the voltammetric signals (three replicas per apple *nalewka*). PC1, PC2 and PC3 explained 98.0% of the total variance between the samples (PC1 75.9%, PC2 16.1% and PC3 6.0%). As observed in the figure, the clusters corresponding to the five studied *nalewka* liqueurs were visibly separated and discriminated. Grey Reinette *nalewka* appears in the positive area of PC2 and Rubin *nalewka* in the negative PC2 region, both clearly separated from the rest of the samples. In turn, Cox Orange appears in the positive PC1 region and Ligol in the positive PC1 area. The repetitions were reproducible with variation coefficients always lower than 0.15 (Table 3).

UV-Visible spectra were used to obtain the CIELab coordinates (L, a*, b*, C, H and BI) giving global color information from the studied samples that was used to discriminate liqueurs by means of PCA. Figure 4b shows that samples could be easily discriminated using the color coordinates. Also, in this case, the variety of Grey Reinette, which has the highest phenolic value, appears clearly separated from the rest. Rubin and Kosztela *nalewkas,* which have phenolic content lower than 700 mg·gallic·acid/L, were located at positive values of PC3 while Grey Reinette, Cox Orange and Ligol with phenolic content higher than 700 mg·gallic·acid/L liqueur appear at negative values of PC3 (PC1, PC2 and PC3 explained 99.4% of the total variance between the samples).

Figure 4 shows the tridimensional PCA scores plot obtained from the Raman signals. PC1, PC2 and PC3 also explained 98.0% of the total variance between the samples (PC1 70.1%, PC2 26.0% and PC3 1.9%). In this case the clusters were larger due to the lower reproducibility of the signals and only Ligol and Grey Reinette could be clearly visibly discriminated according to PC1.

According to the score plots shown in Figure 4, it can be concluded that the e-tongue and the UV-Vis spectroscopy coupled to chemometrics can be used to discriminate *nalewka* samples, whereas Raman spectroscopy could not detect differences between groups.

In order to further quantify the discrimination capability of the three systems, the output of e-tongue, UV-Vis and Raman spectroscopy PCA were used to calculate the Euclidean distance (E. distance)—a variable used to compare the similarity of samples. The smaller the E. distance between two samples, the more similar the taste and flavor [40]. Calculated values of Euclidean distance and coefficients of variation are shown in Table 3. The coefficients of variation (CV) are the ratio between the standard deviation and the mean. It is a measure of the relative variation in observed data, and in this case it applies to points in each group of samples in PCA results.

As observed in the table, the larger Euclidian distances were found in the e-tongue because of the wide range of PC values.

In the case of the e-tongue, Cox Orange-Kosztela and Cox Orange-Rubin *nalewkas* showed the highest value of Euclidean distance, reflecting the large differences in the organoleptic properties observed between those pairs of *nalewkas*. In turn, the similarities in the flavors of Kosztela and Rubin *nalewkas* resulted in a low Euclidean distance.

Euclidean distances calculated from CIELab results showed the highest values of relation between Grey Reinette and other samples. In this case, the same as in e-tongue, results of coefficients of variation indicated low-variance.

On the other hand, the smallest distance was observed between the Rubin and Cox Orange sample when analyzing Raman output. Additionally, distances are comparable in the case of Kosztela and Rubin groups and Kosztela and Cox Orange groups. In comparison to the results of CV obtained from the three techniques in the Raman spectra case, the range of values is wide (0.07–1.13). It should be noticed that CV >1 is considered high-variance.

**Figure 5:**
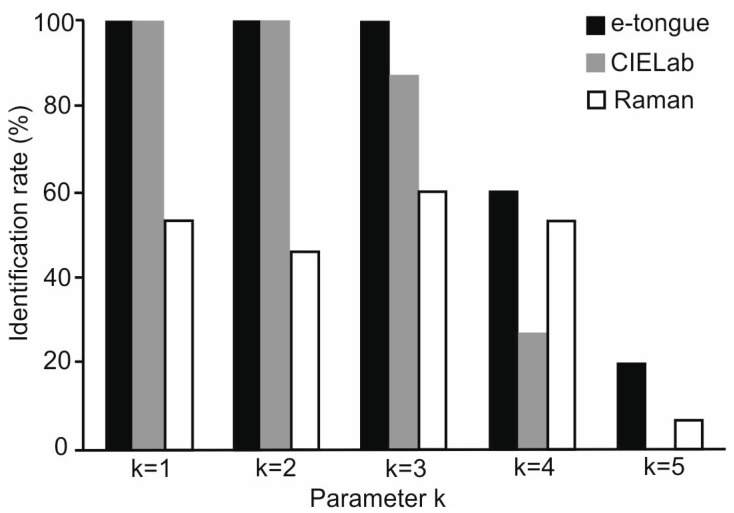
Identification rate of the k-Nearest Neighbors algorithm with different k values.

The kNN is a machine learning technique based on pattern recognition [4]. This classification method is based on a distance matrix, in which an object is classified according to the classes of its K-nearest neighbors in the data space, i.e., it classifies unlabeled objects based on their similarity with samples in the training set. In our case, as there are five samples x three replicas, creation of a test and training sets was impossible, so classification was done for all samples among each other. Testing was done by the program written in Python that implements the kNN classification algorithm using multi-dimensional vectors calculated with Euclidean distance. As observed in Figure 5, the optimal kNN models were obtained using 1 to 3 for nearest neighbors. For electronic tongue, a kNN of 1, 2 or 3 provides a classification accuracy of 100% in all cases, while for CIELab the number of nearest neighbors were 1 and 2, also with a classification accuracy of 100% in both cases. For Raman, the optimal kNN model was obtained using the three nearest neighbors with a classification accuracy of 60%. These results also confirm those obtained with PCA where the clusters corresponding to the five studied *nalewka* liqueurs were visibly better separated and discriminated using the e-tongue and CIELab.

PLS-1 was carried out to establish correlations between signals obtained from the instrumental analysis (e-tongue, UV-Vis and Raman spectroscopy) and chemical parameters (polyphenolic content and the alcoholic degree measured as density). The classification models were subjected to validation by means of the “leave-one-out” method. Therefore, each sample is classified by means of the analysis function derived from the other samples (all cases except the case itself). This process was repeated k times (as many as samples) leaving out one different sample each time—the one to be classified—which acts as the model validation sample. Thus, with this approach, all samples are used once as validation. PLS-1 regression builds a calibration model, incorporating a relationship between sets of predictors and responses based on the structure of signals considering the responses obtained by the corresponding instrumental analysis as the X-variable set and the corresponding chemical parameter as the Y-variable set. PLS-1 models both the X- and Y-variable set simultaneously to find the latent variables in X that will best predict the latent variables in Y. Calibration fits the model to the available data, while validation checks the model for new data. Results are shown in Table 4.

**Figure 6:**
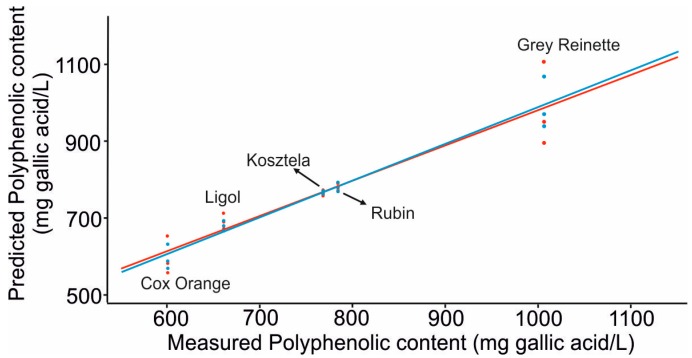
Plot of predicted polyphenolic content obtained with the e-tongue vs. the values of polyphenolic content obtained by the Folin–Ciocalteu method.

Figure 6 shows an example of the linear correlation between polyphenolic content predicted by the voltammetric e-tongue system versus the values of polyphenols obtained by the Folin–Ciocalteu method of the *nalewkas* (calibration in blue and validation in red color).

Good correlations were found between the e-tongue and both the polyphenolic content (0.98 in calibration and 0.93 in validation) and the alcoholic content measured as density (0.93 in calibration and 0.88 in validation) and using four latent variables. As expected, correlations were better with the polyphenolic content because, as already shown in Figure 1, voltammograms show peaks directly related with the phenolic content.

The best correlations with the polyphenolic content were obtained with the UV-Vis data. R^2^ higher than 0.99 in both calibration and validation were found using only three latent variables. In contrast, correlations between CIELab data and density were lower and R^2^ of 0.81 in validation was obtained indicating that this technique is not useful to evaluate the alcoholic content.

Correlations between UV-Vis data and the polyphenolic content showed lower errors in calibration (RMSEc) and prediction (RMSE_P_) than e-tongue and Raman methods. In the case of the density, the errors obtained were similar in all methods although the Raman method provided the lowest error.

Finally, Raman data presented a poor correlation with the polyphenolic content and a good correlation with the density in calibration (R^2^ of 0.96). This can be explained taking into account the contribution of the vibrational modes of the ethanol. The correlation in validation decreased due to the dispersion of the data registered in different repetitions.

These results indicated that the electronic tongue is superior because it shows good discrimination capabilities while providing good correlations with both polyphenolic content and density, whereas the CIELab method is better correlated with polyphenolic content and Raman shows better correlations with density.

## 4. Conclusions

In summary, in this work, the electronic tongue, UV-Vis and Raman spectroscopy combined with chemometric methods have been used to discriminate strong alcoholic liqueurs called *nalewka,* made from different apple varieties: Ligol, Kosztela, Grey Reinette, Rubin and Cox Orange.

The electronic tongue, formed by an array of sensors based on phthalocyanine-modified carbon paste electrodes, could discriminate the five liqueurs prepared from different apple varieties. Analyses are fast and once the system is calibrated measurements take ca. 6 min per sample. Moreover, PLS-1 models showed good correlations between signals of the electronic tongue and the phenolic content and the density (which is related to the alcoholic content) with only four latent variables, correlation coefficients close to 0.9 and low errors. Statistical treatment of CIELab coordinates extracted from UV-Vis spectra also showed a good discrimination capability and excellent correlation with the antioxidant content. However, Raman spectroscopy showed a poor discrimination capability but excellent correlation with the alcoholic content.

Because the electronic tongue showed excellent discrimination capabilities and the best correlations with both polyphenolic content and alcoholic degree, it can be concluded that this technique is the most advantageous for the analysis of high alcoholic beverages such as liqueurs. The lower price, ease of use and portability of the modified electrode system makes it a possible alternative tool to analyze samples in situ.

This study could help to broaden knowledge of these traditional Polish spirit beverages and could be useful for authenticity assessment of food products.

## Figures and Tables

**Table 1:** Phenolic content and density of liqueurs made from different varieties of apple.

Apple Variety	Phenolic Content (mg·gallic·acid/L)	Density (g/cm^3^)
Ligol	661.01	1.1061
Kosztela	767.81	1.0982
Grey Reinette	1005.98	1.0955
Rubin	783.88	1.0946
Cox Orange	600.52	1.0995

**Table 2:** CIELab color parameters of liqueurs made from different varieties of apples.

Apple Variety	L*	a*	b*	C*	h* [°]	BI
Ligol	87.74 ± 0.01	−0.10 ± 0.01	31.36 ± 0.01	31.36 ± 0.01	90.18 ± 0.01	42.39 ± 0.00
Kosztela	87.52 ± 0.01	1.16 ± 0.01	32.45 ± 0.01	32.47 ± 0.01	87.96 ± 0.01	45.49 ± 0.01
Grey Reinette	87.05 ± 0.01	0.41 ± 0.01	48.50 ± 0.01	48.50 ± 0.01	89.52 ± 0.01	76.64 ± 0.02
Rubin	92.52 ± 0.01	−0.34 ± 0.01	28.02 ± 0.01	28.02 ± 0.01	90.69 ± 0.01	34.46 ± 0.01
Cox Orange	92.91 ± 0.01	−1.43 ± 0.01	22.00 ± 0.01	22.04 ± 0.01	93.71 ± 0.01	24.90 ± 0.00

All values given are the mean calculated from three determinations ± SD (standard deviation).

**Table 3:** Calculated values of Euclidean distances and coefficients of variation between groups according to PCA results from e-tongue, CIELab parameters and Raman Spectra.

Relation between Groups		E-Tongue		CIELab Parameters		Raman Spectra	
		E. Distance	CV	E. Distance	CV	E. Distance	CV
Ligol	Kosztela	5899.50	0.06	4.19	<0.01	28.83	0.15
Ligol	Grey Reinette	2808.12	0.15	41.97	<0.01	34.86	0.18
Ligol	Cox Orange	6064.19	0.06	22.81	<0.01	21.88	0.29
Ligol	Rubin	5066.53	0.10	10.60	<0.01	20.34	0.23
Kosztela	Grey Reinette	6514.71	0.05	38.57	<0.01	20.07	0.12
Kosztela	Rubin	3238.44	0.14	13.97	<0.01	8.94	0.07
Kosztela	Cox Orange	10191.66	0.04	26.66	<0.01	8.58	0.36
Grey Reinette	Rubin	5916.40	0.09	51.48	<0.01	19.26	0.13
Grey Reinette	Cox Orange	5115.47	0.07	64.30	<0.01	17.25	0.24
Rubin	Cox Orange	8118.85	0.06	13.17	<0.01	2.13	1.17

E. distance—calculated Euclidean distance between two groups. CV (coefficient of variation)—sum of SD from two groups divided by the Euclidean distance between them.

**Table 4:** Statistical parameters obtained for the PLS-1 regression model established between the chemical parameters and the voltammetric (e-tongue), CIELab and Raman responses towards *nalewka* liqueurs.

**Voltammetric Outputs**					
**Parameters**	**R^2^_C_^(a)^**	**RMSE_C_^(b)^**	**R^2^_P_^(c)^**	**RMSE_P_^(d)^**	**Latent Variables**
Polyphenolic content (Folin–Ciocalteu method)	0.976744	29.75508	0.939679	47.74666	4
Density	0.925237	0.001112	0.878397	0.001751	4
**CIELab Outputs**					
**Parameters**	**R^2^_C_^(a)^**	**RMSE_C_^(b)^**	**R^2^_P_^(c)^**	**RMSE_P_^(d)^**	**Latent Variables**
Polyphenolic content (Folin–Ciocalteu method)	0.996525	8.180929	0.994252	11.27284	3
Density	0.879691	0.001410	0.807301	0.001912	3
**Raman Outputs**					
**Parameters**	**R^2^_C_^(a)^**	**RMSE_C_^(b)^**	**R^2^_P_^(c)^**	**RMSE_P_^(d)^**	**Latent Variables**
Polyphenolic content (Folin–Ciocalteu method)	0.906231	42.49629	0.793365	67.59048	6
Density	0.962399	0.000788	0.856644	0.001650	3

^(a)^ Squared correlation coefficient in calibration; ^(b)^ Root mean square error of calibration; ^(c)^ Squared correlation coefficient in prediction; ^(d)^ Root mean square error of prediction.
